# Response to the impact of COVID-19 by health professions education institutions in Africa: a case study on preparedness for remote learning and teaching

**DOI:** 10.1186/s40561-023-00249-7

**Published:** 2023-05-09

**Authors:** Shalote Chipamaunga, Champion Nestai Nyoni, Mike Nantamu Kagawa, Quenton Wessels, Elliot Kafumukache, Rudo Gwini, Gwendoline Kandawasvika, Patricia Katowa-Mukwato, Rangarirai Masanganise, Rudo Nyamakura, Idah Nyawata, Louise Pretorius, Kefalotse Dithole, Clemence Marimo, Aloysius Gonzaga Mubuuke, Scovia Nalugo Mbalinda, Lynette Jean van der Merwe, Detlef Prozesky

**Affiliations:** 1 Department of Health Professions Education and Student Support, Faculty of Medicine and Health Sciences, University of Zimbabwe, Harare, Zimbabwe; 2 University of the Free State, Bloemfontein, South Africa; 3 Makerere University, Kampala, Uganda; 4 University of Namibia, Windhoek, Namibia; 5 University of Zambia, Lusaka, Zambia; 6 National University of Science and Technology, Bulawayo, Zimbabwe; 7 Cavendish University Zambia, Lusaka, Zambia; 8 University of Botswana, Gaborone, Botswana

**Keywords:** ADKAR framework, COVID-19, Emergency remote teaching (ERT), Formal online learning (FOL), Remote learning and teaching, Undergraduate programme

## Abstract

**Background:**

Africa, like other parts of the world, continuously strives to deliver quality health professions education. These efforts are influenced to a larger extent by the socio-economic and cultural context of the region, but also by what happens globally. The global disruption caused by the COVID-19 pandemic in 2020 necessitated the implementation of emergency remote teaching to continue delivering on the mandate of educating future health professionals. The purpose of this research was to describe the response of selected health professions education institutions in Southern Africa to the impact of COVID-19 and their preparedness for remote learning and teaching.

**Methods:**

A case study design was applied using an adapted ADKAR model as a conceptual framework for data interpretation. The purposively selected study population consisted of educators, students, and administrators in undergraduate medical and nursing programmes from six institutions in five countries.

**Results:**

A total of 1307 respondents provided data for the study. Many of the institutions were caught off-guard when most educators and almost all students were required to leave their universities and go home. Stakeholders immediately became aware of the need to adopt online approaches as an emergency measure. In all programmes, educators, students, and administrators agreed that change was desired, and students realised that they had to take charge of their own learning independently. Overall educators reported confidence in the ability to use of standard Microsoft software, while knowledge of learning management systems proved more challenging for both educators and students. Many stakeholders, especially students and administrators, reported uncertainty about their ability to function in the new reality. Conducive family dynamics, a quiet space to study, good connectivity, a reliable electricity supply and appropriate devices were reported to reinforce learning and teaching.

**Conclusions:**

The findings highlight the need for higher education institutions to prepare for alternative modes to face-to-face learning and teaching approaches with the ultimate aim of transitioning to full online learning more expeditiously. This requires scaling up educational infrastructure, prioritising strategic directives driving continuous professional development of educators and fostering co-constructivist approaches towards student centered education.

## Background

The outbreak of Coronavirus disease 19 (COVID-19) infection became a global pandemic in March 2020 and caused widespread disruption in all spheres of society. Countries had to adopt containment and mitigation measures such as restrictions on the movement of persons and human congregation (WHO, 2020a, b). Planned educational events requiring physical interactions were suspended or cancelled, especially in higher education institutions in Africa, many of which were indefinitely closed (World Economic Forum, 2021). The fast pace of the spread of infection and rise in mortality is portrayed in Table 1. Due to this enormity the restrictions related to COVID-19 were so rapid that contingency measures for continued learning and teaching activities could not be adequately planned.

**Table 1:** Enormity of the pandemic

Country	Covid-19 reported infections		Covid-19 reported deaths	
	30 March 2020	25 October 2020	30 March 2020	25 October 2020
Botswana	4	187,281	1	2407
Namibia	16	128,938	0	3554
South Africa	1586	2,921,886	9	89,163
Zambia	39	209,722	3	3661
Zimbabwe	9	132,954	6	4677

Students and educators were physically isolated and disconnected from mainstream educational settings, where face-to-face instruction was the dominant approach to learning and teaching, especially in the health sciences disciplines (*What Will Higher Education in Africa Look like after COVID-19?*, n.d.). Health professions education institutions in Southern Africa were greatly affected by these measures (Schmutz et al., 2021). Both educators and learners had to find ways of remaining connected and to ensure that some form of learning continued.

Social, economic, and cultural contexts influence health professions education programmes and their adaptation to crisis situations such as the COVID-19 pandemic worldwide—so too in Southern Africa. Health professions education in Southern Africa is predominantly offered in residential universities and colleges using pedagogical approaches which integrate face-to-face and blended classroom teaching and clinical placements (Greysen et al., 2011). Medical and nursing programmes are strongly influenced by the capacity of institutions and the contextual need to produce human resources for health to achieve universal health coverage (Greysen et al., 2011). The majority of the countries in this region are classified as low-income countries with insufficient investment in health and health professions education (Greysen et al., 2011). This lack of training resources explains the limitations experienced in developing comprehensive contingency strategies to cope with disruptions in education processes. Clinical education relies on authentic clinical learning within hospitals and clinics, supplemented by simulation-based education and other innovative pedagogies (AlHaqwi et al., 2014; Liljedahl et al., 2016). The disruptions caused by COVID-19 arguably affected the attainment of competence among students in the health professions in this region (Ossai & Ogbuoji, 2021).

Some health professions education institutions in Southern Africa reported adopting emergency remote teaching (ERT) as a strategy for educators to continue engaging with their students during the COVID-19 pandemic (Greysen et al., 2011). ERT is described as a short-term solution aimed at achieving temporary access to instruction, in a manner that is quick to set up and is reliable during crises which disrupt formally planned educational activities (Hodges et al., 2020). Several authors present compelling evidence that ERT can be a precursor for formal online learning (FOL), which has long been recommended as a strategy for health professions education (Hodgson & Hagan, 2020; Khalil et al., 2020; World Health Organization, 2013). While suggestions for formal online learning have taken on an increased impetus, they are not new: the World Health Organization (WHO), in its publication in 2013 on “Transforming and Scaling up Health Professionals’ Education and Training” recommended FOL as a tool for interprofessional education, mainly if it is delivered in an open-access environment (Khalil et al., 2020). With the advent of the Fourth Industrial Revolution, institutions which had not taken serious measures to migrate to a higher level of technology use would have been aware that they were lagging behind with requirements of modern educational approaches. The COVID-19 pandemic clearly exposed the limited uptake of this recommendation from WHO in the Southern African region—hence ERT being used as a proximal alternative (Gordon et al., 2020; Kagawa et al., 2021).

Since the onset of the COVID-19 pandemic, literature on the adoption of ERT in health professions educational programmes has been emerging rapidly (Mukhtar et al., 2020; Oliveira et al., 2021). Institutions are documenting essential experiences and lessons which are further incorporated into sustainable formal online strategies. However, most of this literature is from developed countries whose recommendations may not be useful within the health professions education context of the Southern African region (Kagawa et al., 2021). There is limited published literature regarding the experiences of ERT from less-resourced settings, such as those from institutions in the Southern African region (Kaliisa & Picard, 2017; Mshana, 2018). We suggest that a description of the awareness, desire, knowledge and ability of stakeholders from these institutions about adopting ERT, as well as factors reinforcing such adoption, is important in winnowing strategies that could be adopted to formalise FOL in the region. In future, health professions education institutions in developing countries are bound to face similar challenges to those outlined in this publication. The purpose of this research was therefore to describe how selected health professions education institutions responded to the impact of COVID-19 and their preparedness for remote learning and teaching.

## Methods

In this study we employed a case study design and adapted the ADKAR framework (Hiatt, 2006; The Prosci ADKAR® Model|Prosci, 2003; Kachian et al., 2018) to formulate the research questions, and analyse and interpret data. The ADKAR framework focuses on stakeholders’ **Awareness** of the need for change; their **Desire** to participate in and support the change; whether they have the **Knowledge** needed for the change, their **Ability** to implement the required behaviour changes; and the **Reinforcement** needed to sustain the change. While the ADKAR framework is designed for managing purposeful organizational change, it was adapted to guide in the assessment of an unplanned change management process, characteristic of emergency situations. Such situations impact on the awareness and desire to change differently compared to planned change. The framework has been used in promoting change management and its influential role of change management on technology adoption has been demonstrated in previous research (Dana et al., 2016; Goyal & Patwardhan, 2018; Lowery, 2010). The stakeholders were educators, administrators and students in the programmes and the study questions refer to them.

### Study questions

1. Were stakeholders **aware** of the need to adjust their teaching and learning approaches? What adjustments did stakeholders make to cope with the disruptions due to COVID-19?
2. Did stakeholders **desire** to participate in and support adjustments for remote learning and teaching?
3. What **knowledge** and skills did stakeholders have to carry out remote learning and teaching?
4. What **abilities** did stakeholders require to carry out remote learning and teaching?
5. What factors did stakeholders perceive to **reinforce** and sustain or obstruct remote learning and teaching?

## Research design

A case study design was applied in this study (Yin, 2009). The case study design allowed the researchers to explore in-depth the complex issues regarding the change process in response to COVID-19 in the real-life setting of the participating institutions in Southern Africa. It also allowed the researchers to acknowledge the various contextual influences in health professions education programmes across the region, appreciating each case as it exists. In Yin’s classification a Type 4 case study design was used which includes multiple units of analysis with multiple cases (Yin, 2009).

### Study population and sampling

The study population consisted of educators, students and administrators in undergraduate medical and nursing programmes from the following institutions: the National University of Science and Technology (Zimbabwe), University of Botswana, University of the Free State (South Africa), University of Namibia, University of Zambia, and University of Zimbabwe. These institutions were purposively selected over a period of two months on the basis of their willingness to participate in the study and share their experiences on their response to the COVID-19 pandemic (Etikan et al., 2016). Within this group, homogeneous sampling was applied by inviting those with nursing and medical programmes to participate.

In general, nursing and medical programs are the most dominant in most health professions education institutions, therefore, the potential for reaching a higher sample during COVID-19 restrictions. Through snowball sampling, additional participants who were purposeful to the study were recommended by their peers (Noy, 2008). Deans, Heads of Departments, and administrators responsible for information, communication and technology (ICT) services were identified as administrators; since teaching and administrative functions may overlap, individuals were allocated to a category based on the main focus of their work. Whole populations of groups in each programme were sampled for the study. This was done since the quantitative data which were also collected for the case studies would provide an opportunity for specific comparisons between programmes. All included participants had to be involved in instituting measures in responding to the disruptions to teaching and learning because of COVID-19 in their programme or institution.

### Data collection

In each institution data collection commenced immediately after ethics clearance was obtained locally.

Data were collected from 1st October 2020 to 31st March 2021. Quantitative and qualitative data were collected using researcher developed questionnaires with structured and semi-structured elements. Separate questionnaires were designed for educators, students and administrators. The questionnaires focused on information regarding the programme, the educational situation before COVID-19 and the institutional response to the COVID-19 pandemic. The adapted ADKAR model determined the structure of questions related to the institutions’ response to the COVID-19 pandemic. Piloting of the questionnaire was carried out using two volunteers from each stakeholder group across all institutions included in this study and adjustments were made based on the pilot outcomes. Potential participants were invited to the study at institution level through an electronic information leaflet which explained the purpose and process of engaging with the study. An option to opt out of the study was presented and implied consent was assumed when participants proceeded to complete the questionnaire. Questionnaires were prepared for online completion and submission; however, in some cases where access to the electronic link to the questionnaire was not feasible, anonymised paper versions of the questionnaire were distributed and data were entered on behalf of the participants into the electronic platform. It is to be noted that during the peak of COVID-19 pandemic, it was difficult and, in some instances, impossible to collect data through any other means.

### Data analysis

Data were analysed through a multi-step deductive process aligned with the adapted ADKAR framework. The researchers checked the data for completeness and made sure all data entries are correct.

1. Researchers in each programme first analysed their data to produce a case study:
  The initial step applied descriptive statistics which captured the frequencies of quantitative responses from the structured questions.
  The next step consisted of open coding to create categories to groups and summaries of the qualitative data. These data provided additional depth to the quantitative data.
  Third summarised qualitative and quantitative data were integrated using the framework shown in Table 2 below, and through triangulation, themes were produced for each element of the adapted ADKAR model for each programme. The summarised data were presented as a complete case study.
2. The final step of analysis amalgamated the cases from each programme and institutions across the entire region under the elements of the adapted ADKAR model of change, again using the framework shown in Table 2. In this way a collective case study was developed integrating all the individual cases.

**Table 2:** Data analysis framework

1. The nature of the programmes 1.1 Medical/ Nursing 1.2 Number of staff, students, and administrators in the programme
2. Baseline situation 2.1 How teaching was done before the lockdown
3. Awareness of the need for change 3.1 How National and University policy brought about an awareness on the part of stakeholders that they had to change the way they work
4. Desire to participate in change 4.1 How stakeholders decided to play their part in managing the new reality 4.2 How learning, teaching, and support actually changed
5. Knowledge and skills needed 5.1 Stakeholders’ knowledge about and experience in using tools they had to use to manage the new reality
6. Ability to implement change 6.1 The self-perceived ability of stakeholders to manage the new reality
7. Reinforcement to sustain change 7.1 Resources available to stakeholders to work in the new reality 7.2 Factors supporting and sustaining the change to working in the new reality 7.3 Factors hindering the change to working in the new reality

### Ethical approval and consent to participate

For each institution ethical clearance was obtained from its own institutional review board or committee, having provided the information required in each situation. The principles of the Belmont report of 1979 and the International Conference on Harmonization of 2002 were applied to safeguard the participants (Biomedical & Research, 1978; Dixon, 1999). All participants received information related to the study and participation was voluntary. Anonymity and confidentiality were maintained throughout this study.

### Trustworthiness

Elements of trustworthiness were applied in this study (Biomedical & Research, 1978). We attempted to enhance the credibility of our research by triangulating data within each programme, and triangulating the themes obtained between stakeholders. We also communicated regularly between ourselves to standardise data collection and analysis. Our decision to involve the dominant programmes in six southern-African institutions in the research should enhance the transferability of the findings to other health professions education programmes in different contexts. We paid attention to the dependability of the research by rigorously documenting our discussions and decisions in detailed minutes of our fortnightly meetings. Finally, we tried to ensure conformability by designing written guidelines to guide each step of the research process, thereby illustrating how we reached our conclusions.

## Results

A total of 1307 respondents provided data for the study from eleven progammes at six institutions in five countries (Tables 3, 4).

**Table 3:** Programmes and countries

Country	Programmes		Total
	Medical	Nursing	
Botswana	1	1	2
Namibia	1	1	2
South Africa	1	1	2
Zambia	0	1	1
Zimbabwe	2	2	4
Total			11

**Table 4:** Respondents

Programmes	Educators	Students	Administrators	Total respondents
Nursing	102	518	28	1307
Medical	116	543		

The results in this section will first provide an overview of the pre-COVID-19 educational approach at each programme and institution, followed by the elements of the adapted ADKAR model in sequence to reflect responses to the COVID-19 pandemic. The factors under each of the five elements which represent the milestones programmes should achieve for change to be successful build on each other and are interrelated.

### The pre-COVID-19 situation

Most nursing programmes lasted four years and most medical programmes lasted five years. Curricular models varied across programmes, with the traditional model of preclinical teaching for two years followed by clinical teaching predominating. Learning and teaching were reported to take place face-to-face, integrating laboratory and clinical work across all the institutions. Some institutions informally used blended learning approaches through established learning management systems; however. Most institutions did not use electronic platforms to enhance learning. All summative assessments were reported to be conducted face-to-face, including direct observation in the clinical environments. Administrators reported regular meetings with programme stakeholders to identify problems and offer support in solving them.

### The response to COVID-19

The response to COVID-19 describes approaches used by the programmes and institutions in response to the pandemic and related restrictions from the viewpoint of educators, students, and administrators across the five institutions included in this study.

### Awareness

Awareness of the need for change evolved as the pandemic unfolded. All three groups of stakeholders became immediately aware of major changes, when national regulations obliged most educators and almost all students to leave their universities and go into isolation at home. Some staff members were allowed to continue working from their offices but with strict measures to limit disease transmission. Over time these measures were relaxed piecemeal to varying degrees in different programmes, with students eventually being allowed to return to their universities under strict control measures:

Staff members are expected to continue teaching as usual but with measures to prevent transmission (e.g., masking, social distancing, hand hygiene) strictly enforced (Educator).

With lockdown, academic programmes were suddenly disrupted, and educators realised the need for remote teaching. Students realised that they had to do the best they could to continue learning at home, while waiting for guidance from their programme administrators. Administrators realised that they had to work online now, giving additional support to educators and students:

We have been working under pressure covering a learning workload of 9 months in 3 months. We have been having ward rounds for reduced period when we open school for the reduced time and our weeks for ward round rotations have been halved. More than 80 percent of lectures have been delivered online (Student).

The transition to online teaching did not occur immediately. For the first time educators were expected to work remotely, and this changed their approach to teaching. They became aware that they had to adopt online approaches and tried to use whatever method available or known to them to teach the subject content—including social media platforms (WhatsApp and Facebook), business communication platforms (Microsoft Teams and Zoom), learning management systems (Blackboard, Moodle and Google Classroom), and narrated lecture PowerPoint slides along with audio recordings of questions and answers. For many, this would be an adjustment with a steep learning curve, immediately and in future:

COVID -19 has changed the Education Industry – A lot of new innovations will take place. Universities need more funding to support the students and staff to use this technology in the near future. (Administrator)

Online teaching is the way to go and should not be stopped even after the COVID-era. (Administrator).

Educators and students also became aware that pandemic-related restrictions would severely hamper clinical teaching. In addition, educators and students soon became aware of severe limitations that would be imposed by poor connectivity, especially in rural communities, the cost of internet packages, and unsuitable communication devices:

After the initial hard lock down with students and staff at home, some students were allowed to return to university. However, the academic year was then markedly shortened and clinical rotations had to be condensed. Due to restrictions on clinical platforms, we had to move to more simulated and paper-based teaching rather than teaching in the wards and clinics with actual patients. The same applied for assessments (Educator).

### Desire

Desire to support change was assessed in terms of whether participants perceived they should support their programmes in the new reality, and the changes in practice that this desire brought about. In all programmes, educators, students, and administrators almost universally agreed that they should change and wanted to change:

The desire to deliver some teaching to students drives me forward … (Educator)

Apply personal effort to the best of my abilities…. Find alternative means in which I can successfully cover most the learning materials given the curriculum (Student).

Educators wanted to change from face-to-face to online teaching which they also wanted to master. They would offer online teaching synchronously or asynchronously as circumstances demanded and only conduct face-to-face practical teaching when it could be done safely, supporting students with the resources they needed:

The crisis became an opportunity for me to learn and sharpen my skills on online teaching. An opportunity to help support students that were resisting change and help them change their mindset and progressed well. (Educator).

Students made it clear that they wanted the educational changes to work for them so that their studies could succeed—rather passively in some cases, looking for guidance from educators, but in several instances realising that they had to take charge of their own learning independently. Administrators in some institutions wanted to provide increased support online to staff and students, to plan for increased ICT training and adequate connectivity and where possible to extend counselling services:

I am available to staff members to discuss their needs and challenges (Administrator)

Changes in practice resulting from a desire to change were broadly similar across programmes despite individual differences and strengths. Educators reported that their teaching changed completely or partly with the widespread use of relevant and available online platforms and software, and clinical teaching adapted for safety. Theory teaching became almost entirely remote with materials being provided online or by email. Theory assessment was also done online, using tools like open book exams, assignments, and participation in discussions. During strict lockdown, clinical and skills teaching was limited and focused on relevant theory using case studies and demonstration videos. Skills assessment had to be limited to their theory component, with some innovations like assessing student videos. Forced by circumstances individuals had to adapt, but there was a strong desire for face-to-face teaching and assessment to resume their proper place as circumstances permitted. Students similarly reported major changes in learning with online learning for theory predominating and student-led use of social media, search engines, and peer support.

Treat online learning as I would face to face teaching and equip what's missing with e.g. You Tube learning videos (Student)

Students, too, experienced reduced clinical and skills learning and assessment. The ‘Desire to support change’ was demonstrated to be remarkably similar among all programme stakeholders across all institutions.

### Knowledge

According to the ADKAR model, knowledge on how to change is important for change to take place. Assessing existing knowledge is recommended because the size of the knowledge gap may impact the success of the adoption of the desired change. This study asked participants to rate themselves on their confidence in implementing the adjustments and their experience in the relevant tools to be used (Tables 5, 6, 7).

**Table 5:** Reported educator and student confidence in using software needed for ERT

Tool	Reported confidence	Educators % (N = 218)	Students % (N = 1061)
Microsoft Word	Very confident	91.2	66.5
	Some confidence	8.3	31.5
	Not at all	0.5	2.0
Microsoft PowerPoint	Very confident	91.2	59.7
	Some confidence	8.8	37.1
	Not at all	0	3.2
Search engines (e.g. Google, Firefox)	Very confident	79.7	72.7
	Some confidence	19.8	24.5
	Not at all	0.5	2.8

**Table 6:** Reported educator and student experience of tools that support ERT

Tool	Reported use	Educators (N = 218)	Students (N = 1061)
WhatsApp	Often	32.9	37.0
	Sometimes	47.5	47.5
	Never	19.6	15.5
Zoom, Skype, BlueJeans	Often	40.8	41.7
	Sometimes	36.4	41.5
	Never	22.8	16.7
e-platforms (Moodle, Teams etc.)	Often	55.0	45.3
	Sometimes	26.5	24.2
	Never	18.5	30.4

**Table 7:** Reported administrator confidence in using tools which support ERT

Tool	Reported use	% (N = 27)
Microsoft Office	Very confident	55.6
	Some confidence	33.3
	Not at all	11.1
Zoom, Skype, BlueJeans	Very confident	33.3
	Some confidence	55.6
	Not at all	11.1
e-platforms (Moodle, Teams etc.)	Very confident	33.3
	Some confidence	63.0
	Not at all	3.7

It was evident that respondents were aware of the knowledge and skills needed to implement ERT and learning as a potential impediment to remote learning and teaching:

Lecturer incompetence with ZOOM (Student).

Overall educators in all programmes reported high confidence levels with the use of commonly used tools like Microsoft Word, PowerPoint and e-mail; confidence with search engines was reported to be lower. Educators recognised WhatsApp as a useful tool for ERT but their experience with it was limited; so too for Zoom, Skype and Teams and common e-learning platforms. For all these tools a diverse skill range was reported between programmes.

I have some knowledge of some of the teaching platforms and I should manage with some support from the University (Educator).

Overall, students’ reported confidence in using Microsoft programmes was lower than that of educators but their confidence in using search engines was the same (Tables 5, 6). Students’ reported experience of tools like WhatsApp and Zoom resembled that of their educators but many had no experience of platforms like Moodle and Teams. Administrators reported general competence in the tools they needed to support the programme.

### Ability

In this study, we regarded Ability as the transition from knowledge to action. Many factors that impact this process include resources, physical and intellectual capability, time and psychological blocks, and fears (The Prosci ADKAR® Model|Prosci, 2003). Stakeholders’ ability to implement new skills and behaviours required for ERT was determined in several ways. Their confidence in the use of relevant software and platforms was assessed (Tables 5, 7) and revealed varying levels of ability. Stakeholders were also asked to report on their overall ability in relation to ERT (Table 8).

**Table 8:** Reported stakeholder ability to perform in ERT

	Completely %	Partially %	Not at all %
Educators’ reported ability to conduct teaching in ERT (N = 161)	48.4	45.3	6.2
Students’ reported ability to learn effectively in ERT (N = 947)	14	73.5	12.5
Administrators’ reported ability to support ERT (N = 23)	13	87	0

Educators reported almost equal levels of complete and partial ability to teach remotely, and few felt completely unable to do so. On the other hand, students and administrators reported low levels of complete ability to manage ERT, while 14% of students reported no ability at all.

The overall ability of all three stakeholder groups to implement ERT (Table 8), was mostly positive or partly so. However, given the gaps in confidence in using software and other tools (Tables 5, 7) it is not surprising that ability was reported to be partially or wholly lacking in some cases.

### Reinforcement

This section of the results focuses on the structures across the programmes included in this study that were found to reinforce or constrain the adoption and maintenance of ERT. Educator and student assessments are given in Table 9.

**Table 9:** Educator and student assessment of resources available at home for successful ERT

Requirement	% positive responses	
	Educators (N = 218	Students (N = 1061)
Reliable electricity at home	81.7	60.4
Private space available to work at home	75.2	53.4
Adequate time to do academic work at home	79.4	56.2
Good connectivity at home	70.2	39.0
A computer to use at home	88.5	69.4
A smartphone to use at home	85.3	88.6
Teaching/ learning materials needed are available at home	67.9	46.1
Technical support available at home	52.3	

There were clear differences in educator and student assessment of the resources that would enable them to carry out ERT successfully in a home setting: educators reported having considerably more of these resources available.

For educators devices such as computers and smartphones were mostly available, as was reliable electricity. Most reported having adequate time and private space for their academic work; adequate connectivity and availability of teaching materials less so. In qualitative comments educators reported reliable internet access as a key factor in supporting the change to ERT—many had access either at work or at home, but this was variable; most could afford the data required. The support given by university structures (e.g., libraries) was noted and appreciated but only half reported that their ability to find technical support was adequate. The support and dedication of students and their families was noted as a key element of support for ERT.

Student perceptions of reinforcing and limiting factors were varied. Two highly valued reinforcing factors were conducive family dynamics and a quiet space in which to work undisturbed at home but only about half had this privilege. Internet access was a challenge for many, in terms of electricity supply, connectivity and cost:

I have to walk quite a long distance to my uncle’s house where there is better connectivity (Student).

Although I am privileged enough to have been able to attend online school, more than 75% of the class was not able to because of a lack of resources and connectivity. However, the teaching continued without them and that was unfair (Student).

Having appropriate devices was perceived as essential. Most reported having access to smartphones but noted their limited functionality; computer ownership and access were much more problematic:

I use my phone to read, and the words will be small (Student).

I have to share the laptop with my 3 siblings (Student).

In qualitative comments students’ ability to use relevant online platforms for ERT was noted to be a reinforcing factor, although lack of sufficient time to learn in the ‘new normal’ was mentioned as a constraint. Students appreciated online learning materials provided, for example recorded lectures and voiceover PowerPoints, as well as other forms of material and moral support from their programmes:

Due to several challenges (electricity, data costs, and home environment) not all the students are able to take part in synchronous learning. Learning material is then sent on WhatsApp and Google Classroom and the students can interact with it when circumstances allow (Educator).

In some cases, institutional support was perceived not to be sufficient at all, notably due to poor communication. Moral and practical support from families, community members and senior students was mentioned as a reinforcing factor for ERT, so that in some cases student attitudes were remarkably positive:

All I want to be is the best. While others are complaining of the situation, I know I can spread my wings and fly high up their heads. I am competing at a global scale so whenever I study, I think of that other student in Europe who has everything at his disposal but still I want to make a better doctor than him (Student).

Administrators’ assessment of the institutional resources needed to sustain ERT successfully was largely negative (Table 10).

**Table 10:** Administrators’ assessment of institutional resources for ERT

Resource	% positive responses (N = 28)
Institution’s staffing level is sufficient to support ERT	57.1
Instruction’s budget sufficient to support ERT	14.3
Institution is flexible enough to accept innovations needed for ERT	53.6
Accrediting professional bodies will accept changes due to ERT	60.7
Institution’s bandwidth is sufficient to cope with ERT	25.0
Institution’s hardware is sufficient for ERT	25.0
Institution’s software is sufficient for ERT	35.7
Institution’s library services are geared for ERT	35.7
Institution can provide psychosocial support to students during ERT	35.7

Budgets were particularly judged to be inadequate—so too the bandwidth and hardware needed. More than half of administrators assessed library service support, software availability, staffing levels and the psychosocial support available to students to be inadequate.

Lack of serious support from the parent institution to provide preventive measures in the school……. Lack of moral support to staff who record cases of Covid 19 positive *[sic]*…. and also lack of disclosing the results of students and or academic staff who test positive (Administrator).

Assessment of institutional and professional body flexibility to accommodate ERT was somewhat more positive but still not high. On the other hand, administrators valued the skill, enthusiasm, and commitment of staff and students who pulled together to make the new learning system work.

## Discussion

In this study, we set out to reveal how selected health professions education institutions in the Southern African region had adjusted their academic programmes to cope with the COVID-19 related pandemic. The synthesis and interpretation of the findings was done through the lens of the ADKAR model, which Prosci® describes as a relevant model for describing and informing change within organisations (Hiatt, 2006). Overall, the institutions included in this study adjusted their nursing and medical programmes in response to the COVID-19 pandemic. These reported programmes and institutional adjustments influenced the structure and implementation of their educational programmes.

All the included institutions and programmes reported an awareness of the need to adopt ERT for learning and teaching during the COVID-19 pandemic. This awareness was brought about by the national responses to the COVID-19 pandemic, which restricted physical and social gatherings as well as limited infrastructure and policy for formal online learning (Mulenga et al., 2021). Furthermore, all institutions were expected to continue learning and teaching during this time with online and electronic platforms (including social media) being a proximal emergency solution. Yusuf highlighted the need for continued education, especially for health professions, to supplement the health professionals at the coalface of the COVID-19 pandemic (Ifijeh & Yusuf, 2020). Positive awareness of the need for change is an essential initial aspect of the change process, creating opportunities for sustained change.

The desire to change is associated with a personal decision to change and engage with the emergency remote teaching. The students, educators, and administrators in this study reported a strong desire to engage with the adoption of emergency remote teaching as part of educational strategies during the COVID-19 pandemic. This desire may have been related to the abrupt implementation of containment measures and the drive for continued education. Prosci® explains that the desire to change is enhanced in the change process when the alternative of not implementing the change is worse, when the fear of penalty is high or when there is a desire to belong (Hiatt, 2006). In this case, failing to complete an academic year would have had dire consequences for students, educators, and administrators. Students would not graduate in time, with implications such as financing, while educators might have increased workloads since they would have to cope with students with backlogs in addition to their normal programmes. The health delivery system, which is in dire need of health professionals, would suffer from a truncated production pipeline of health professionals. Whether this desire and the changes it engendered would result in long-term change is still open to question.

Stakeholders in this study appear to have fair but varying knowledge on how to adopt ERT and how to perform within the ERT-related platforms. Prosci®, mentions that knowledge is only effective in adopting change when there is established awareness and desire related to the change (Hiatt, 2006). In this study, most of the knowledge reported by the included stakeholders was based on prior experience of engaging with learning platforms outside an emergency teaching atmosphere. True to the definition of ERT there would not have been sufficient time for formal learning and training related to the implementation of ERT resources (Schlesselman, 2020). Some institutions, through their administrators, had instituted strategies aimed at supporting the knowledge of educators and students regarding ERT through training and education. Levett-Jones mentions that a well-established awareness and desire is fundamental in driving self-directed learning approaches among individuals in developing their own knowledge (Levett-Jones, 2005). This self-directedness may have been essential in developing the knowledge of educators who may not have had opportunities for formal institution-based education and training.

Interestingly educators and students were found to have similar knowledge and experience of software needed for learning, thus bucking the trend where students tend to be more skilled than their educators. However, many educators and students report being only partly skilled or not skilled at all. Ferri, Grifoni, and Guzzo attest that pedagogical challenges were embedded in their adaptation to remote teaching in Italy (Ferri et al., 2020). These pedagogical challenges referred to the lack of digital skills of some educators and students. Educators and students within Southern Africa need support on education-related software to enhance pedagogy within electronic platforms within the remits of their universities. Consequently, some institutions in the Southern African region appear to have a dedicated staff for such educator and student support; however, this is not the same across all institutions included in this study. Self-paced electronic platforms where educators and students can access and learn about learning and teaching software are essential in the African context.

Prosci® defines ability as the demonstration of the required changes, which in essence is the translation of knowledge into performance (Hiatt, 2006). In this study, the ability of the included stakeholders was drawn from self-reported data and not the direct observation of performance. Evans et al. in their argument for feedback in education, explain the limitation of self-assessment, where self-assessment outcomes are usually not correlated to actual performance—where individuals often rate their performance higher than their actual performance: the so-called Dunning-Kruger effect (Evans et al., 2002; Kruger & Dunning, 1999). Nevertheless, the participants in this study rated their abilities to apply ERT and learning within their own academic milieu highly, since there was limited institutional support. The literature explains that sufficient time, practice, access to the appropriate tools, including the presence of a coach or role model, are essential elements in fostering the ability to engage with change (Hiatt, 2006).

A generic challenge across many higher education institutions globally relates to adequate resources for learning and teaching influencing strategies towards the reinforcement of adoption of FOL. In this study, resources for learning and teaching were examined as potential strategies for reinforcing approaches for remote learning and teaching. A higher proportion of the educators claimed to have a constant electricity and internet supply while this was not the same for students. Devices for learning and teaching and adequate learning spaces were not optimally available for students, and institutions appear not to have sufficiently budgeted for this change. These findings are similar to those reported by Ferri et al. (2020), where technological challenges appear to be a common challenge in implementing online teaching even in technologically advanced countries like Estonia. Even so the data clearly indicate that significant steps have been taken in these institutions towards integrating FOL more fully into their programmes.

## Recommendations

Based on the findings of this study we argue for concerted efforts at the regional and institutional level that may enhance quality and equity of the educational experience of all health professions students.

In terms of practice, we recommend that institutions should arrange for remedial opportunities for those students that missed learning because of the lockdown. Educational institutions like those in Southern Africa should consider consolidating efforts in devising strategies to enhance remote learning and teaching. Such efforts could include improving learning and teaching infrastructure, zero-rating institutional websites, and providing limited amounts of free data for personal devices, and sharing learning materials across institutions. Institutions with advanced faculty development programmes may be essential in developing shareable programmes for the Southern African context. Co-constructivist approaches could also be embedded in learning and teaching, further allowing students to have a voice in how they are taught within the electronic space.

At the policy level, institutional strategic directives should prioritise continuing professional development for educators as well as strengthening development and investment in educational infrastructure which supports online learning and teaching. Exemplary educational practice should be recognised and rewarded.

In terms of research, there is need to evaluate further the ability to implement ERT by educators, students, and administrators within this setting. The research should focus on evaluating the impact of remote learning and teaching on health professions education students in the Southern African region. Outcomes from such research may be useful in the design and implementation of purposive induction or mentorship programmes for graduates who may not essentially be competent or workplace ready.

### Study limitations

The participation across all institutions included in this study was low—during the COVID-19 pandemic there were restrictions to data collection methods and online platforms were not widely available. As a result, this study’s results are not intended for generalization across the continent or programmes. Similarly, the original intention was to include case studies from fifteen programmes in eight participating institutions, but this proved not to be possible due to challenges faced with data collection and reporting within the timeframes agreed upon by the research team. The results presented in this collective case study therefore exclude findings from four programmes.

## Conclusion

Health professions education institutions in the Southern African region have attempted to adjust their educational programmes in response to COVID-19. These adjustments were affected by limited resources, limited educator competencies, and students’ challenging home environments. Inasmuch as the need to complete the academic year appears to be a proximal justification for the adoption of ERT the impact of the quality of such approaches on the integrity of training of healthcare graduates from these institutions needs further investigation. Educational institutions should ensure that competence is not compromised in an already poorly resourced and heavily burdened health delivery system in Southern Africa.

We are of the opinion that valuable information expressed in this study, including the conclusions that we drew, may well resonate with many institutions and individuals in the continent and that recommendations from this study may be of use.

## Data Availability

The datasets generated during and/or analysed during the current study are available from the corresponding author on reasonable request.

## Abbreviations

ADKAR: Aware, desire, knowledge, ability, reinforce framework
COVID-19: Coronavirus Disease 19
ERT: Emergency remote teaching
FOL: Full online learning
ICT: Information, communication and technology
IRB: Institutional Review Board
WHO: World Health Organization
